# Wnt5a Signaling in Cancer

**DOI:** 10.3390/cancers8090079

**Published:** 2016-08-26

**Authors:** Marwa S. Asem, Steven Buechler, Rebecca Burkhalter Wates, Daniel L. Miller, M. Sharon Stack

**Affiliations:** 1 Integrated Biomedical Sciences Program, Department of Chemistry and Biochemistry, Harper Cancer Research Institute, University of Notre Dame, South Bend, IN 46617, USA; Marwa.S.Asem.2@nd.edu; 2 Department of Applied and Computational Mathematics and Statistics, Harper Cancer Research Institute, University of Notre Dame, Notre Dame, IN 46656, USA; buechler.1@nd.edu; 3 Department of Pathology and Laboratory Medicine, University of Kansas Medical Center, Kansas City, KS 66160, USA; rwates@kumc.edu; 4 Department of Pathology, The Johns Hopkins University School of Medicine, Baltimore, MD 21287, USA; miller.climb@gmail.com; 5 Department of Chemistry and Biochemistry, Harper Cancer Research Institute, University of Notre Dame, South Bend, IN 46617, USA

**Keywords:** Wnt5a, non-canonical Wnt signaling, frizzled (Fzd), receptor tyrosine kinase-like orphan receptor (Ror), cancer, ovarian cancer, senescence, inflammation, microenvironment, epithelial-mesenchymal transition (EMT)

## Abstract

Wnt5a is involved in activating several non-canonical WNT signaling pathways, through binding to different members of the Frizzled- and Ror-family receptors. Wnt5a signaling is critical for regulating normal developmental processes, including proliferation, differentiation, migration, adhesion and polarity. However, the aberrant activation or inhibition of Wnt5a signaling is emerging as an important event in cancer progression, exerting both oncogenic and tumor suppressive effects. Recent studies show the involvement of Wnt5a in regulating cancer cell invasion, metastasis, metabolism and inflammation. In this article, we review findings regarding the molecular mechanisms and roles of Wnt5a signaling in various cancer types, and highlight Wnt5a in ovarian cancer.

## 1. Introduction

Wnt signaling represents a group of signaling pathways that regulate a diversity of processes fundamental to normal development, including cell proliferation, differentiation, polarity, adhesion and motility [1,2]. In mice and humans, 19 secreted lipid-modified Wnt-family glycoproteins have been identified. These Wnt ligands can bind to several receptors including Frizzled family receptors (Fzd), receptor tyrosine kinase-like orphan receptor family (Ror), low-density lipoprotein receptor-related protein co-receptors (LRP) and the related to receptor tyrosine kinase (Ryk) receptor. Due to the large variety of Wnt ligands and receptors, multiple downstream pathways are activated. These pathways can be divided into two main categories: (1) canonical WNT signaling, which is well-characterized and is dependent on the transcriptional activity of β-catenin and (2) non-canonical Wnt signaling that does not involve the transcriptional activity of β-catenin and can be further sub-classified into different pathways [2].

Wnts bind to Fzd receptors and LRP5/6 co-receptors to form a complex that initiates canonical Wnt signaling. In absence of Wnt binding, most cellular β-catenin is bound to the cytoplasmic domain of the cell adhesion molecule E-cadherin wherein it functions to stabilize adherens junctions. Free, cytoplasmic β-catenin is controlled by a degradation complex that comprises Axin, adenomatous polyposis coli (APC) and glycogen synthase kinase 3β (GSK3β). This complex phosphorylates free β-catenin in the cytoplasm and targets it for degradation through the ubiquitin-proteasome pathway [3]. Activation of canonical Wnt signaling results in the recruitment of Axin and Dishevelled (Dvl) to the plasma membrane, and disruption of the formation of the β-catenin degradation complex, leading to accumulation of β-catenin in the cytoplasm, which then translocates to the nucleus, where it binds to T-cell factor (TCF)/lymphoid enhancer factor (LEF) family and activates the transcription of canonical Wnt target genes [3]. In contrast, non-canonical Wnt signaling is independent of β-catenin transcriptional activity and can be further classified into pathways that are less characterized relative to canonical Wnt signaling [4]. Furthermore, it is well established that non-canonical Wnt ligands can antagonize the functions of canonical ligands, inhibiting canonical signaling [5].

Wnt5a is a highly evolutionary conserved non-canonical Wnt ligand that plays a critical role in regulating planar cell polarity (PCP), convergent extension (CE), and epithelial-mesenchymal interaction during embryonic morphogenesis [6]. Homozygous Wnt5a knockout mice show perinatal lethality, due to developmental defects [7]. The aberrant activation or inhibition of Wnt5a signaling is emerging as an important event in tumorigenesis, exerting both oncogenic and tumor suppressive effects [8]. While Wnt5a has been shown to inhibit cell growth, migration and invasiveness of thyroid and colorectal cancer cells [8,9,10], increased Wnt5a expression is involved in aggressiveness of other types of cancers such as melanoma and gastric cancer [11,12]. The tumor suppressor effects that Wnt5a can exert may be achieved through antagonizing canonical Wnt signaling, resulting in inhibition of cell growth and migration [13]. Wnt5a activates various downstream signaling pathways and, in spite of being identified as a non-canonical ligand, it can, under certain circumstances, signal through canonical Wnt signaling pathways [13]. Among the pathways downstream of Wnt5a, PCP and the Wnt/Ca^+2^ pathway are the best characterized.

PCP is the polarizing of epithelial cells within the plane of the epithelial layer [14]. Although there are other activators of PCP, Wnt5a can activate the PCP pathway by forming a complex with Fzd and Ror2 receptors, activating DVL, which in turn activates Rho-family small GTPases, including RhoA and Rac, and their downstream effectors, Rho-associated protein kinase (ROCK), the actin-binding protein, Filamin A and c-Jun N-terminal protein kinase (JNK) [14,15,16]. The activation of PCP leads to remodeling of the actin cytoskeleton, thereby regulating polarized cell morphology and migration within tissues [17]. Furthermore, the Wnt5a-Ror2-Filamin A-JNK pathway mediates the formation of filopodia, an event crucial for Wnt5a-induced cell migration and stimulates lamellipodia formation and reorientation of the microtubule-organizing center (MTOC), processes that are essential for polarized cell migration [18,19]. Wnt5a activation of the Wnt/Ca^2+^ pathway results in the mobilization of free intracellular calcium that regulates many cellular processes, including actin cytoskeleton remodeling and cell motility through activation of calcium-dependent signaling molecules, such as calmodulin dependent protein Kinase II (CAMKII) and protein kinase C (PKC) [20,21,22,23,24].

**Figure 1:**
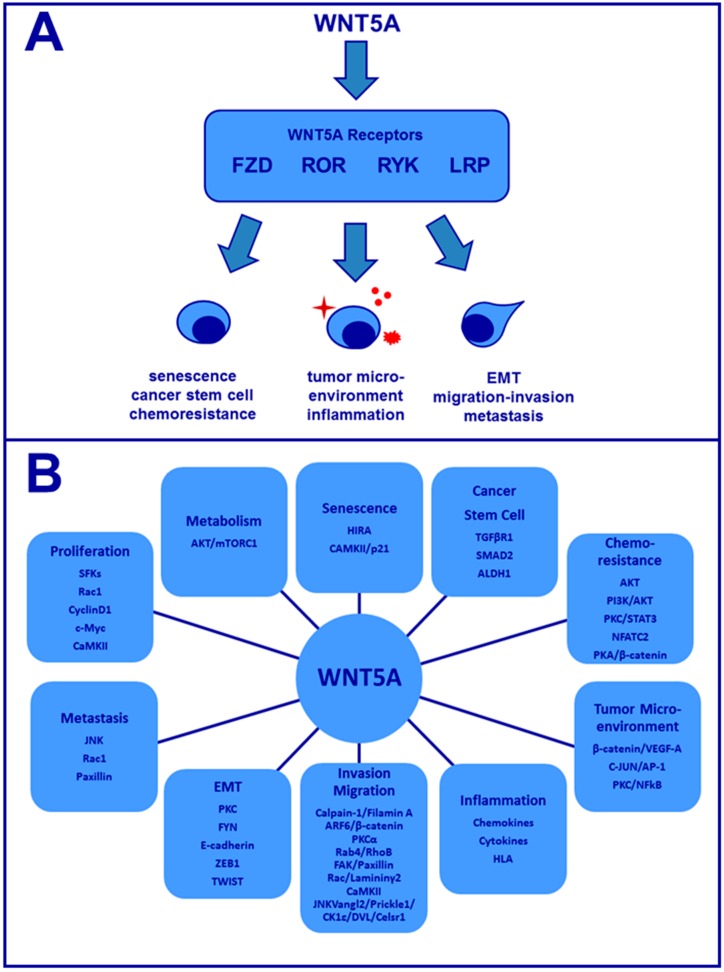
Overview of Wnt5a signaling in cancer. (**A**) Wnt5a binds to a number of distinct trans-membrane receptors to influence a diverse array of tumor cell behaviors; (**B**) Summary of Wnt5a effector proteins that influence cell behavior.

Emerging data support a contribution of specific Wnt5a downstream signaling pathways to cancer development and progression. Furthermore, the diversity of Wnt5a binding receptors and the cell/tissue of origin also likely contribute to triggering distinct signaling pathways (Figure 1A,B) [8]. Another factor that may play a role in regulation of Wnt5a-activated signaling is the presence of a specific Wnt5a isoform within the tissue. The *WNT5A* gene encodes two distinct transcripts expressed from alternative transcriptional start sites leading to production of two distinct Wnt5a proteins, a long 337 amino acid isoform (Wnt5a-L) and a short 319 amino acid isoform (Wnt5a-S).

While the two Wnt5a protein isoforms have no differential effects on activating Wnt/β-catenin signaling, they exhibit different effects on cell proliferation. Interestingly, the Wnt5a-S isoform promotes cell proliferation while the Wnt5a-L isoform inhibits cell proliferation [25]. While these isoforms have been reported with differential expression in breast and cervix carcinomas and neuroblastomas [25], additional studies are needed to determine the mechanism by which isoform expression contributes to oncogenesis.

While Wnt5a is highly expressed in various tissues and organs during embryo development, its expression level in general decreases in adult tissues [17,26]. The exact role played by Wnt5a in cancer remains controversial, as it shows both tumor-suppressing and oncogenic effects in different cancer types. Here, we will discuss recent findings regarding the molecular mechanisms and the emerging roles of Wnt5a signaling in various cancer types.

## 2. Wnt5a and Cellular Senescence

Senescence is the arrest of cell growth, which can be caused by critically shortened telomeres, activated oncogenes such as HRas, DNA damage, and certain cancer therapeutics such as cisplatin [27,28]. Experimentally, there are different markers and morphological features that can be used to detect senescence, including senescence-associated β-galactosidase activity (SA-β-gal), senescence-associated heterochromatic foci (SAHF), promyelocytic bodies (PML), modified chromatin and a large flat cellular morphology [29]. Inducing senescence in cancer cells is a new therapeutic strategy that can be achieved through reactivation of p53, inhibition of CDKs or inhibition of oncogenes such as Myc [30,31,32,33]. The correlation between Wnt5a signaling and senescence was demonstrated for the first time in ovarian cancer [34]. In this study, Wnt5a reconstitution in primary human ovarian surface epithelial cells, the epithelial ovarian cancer cell line OVCAR5 and an in vivo model induced cellular senescence through increasing recruitment of histone repressor A (HIRA) into PML bodies, a main mechanism in senescence, and this process was independent of p53 and p16^INK4a^ [34].

While replicative senescence is irreversible, recent studies show that other types of senescence such as oncogene-induced senescence and therapy-induced senescence are reversible through the senescent-like stress response [35]. A recent study highlighted a novel role for Wnt5a in inducing the senescent-like stress response [36]. In melanoma, Wnt5a-high expression is correlated with aggressiveness of the disease [11,37]. Treating Wnt5a-high cell lines with the chemotherapeutic agent PLX4720 or ionizing radiation showed a significant increase in SA-β-galactosidase, H3K9Me chromatin marks and PML bodies, relative to Wnt5a low cells. In these cells, Wnt5a-induced senescence was driven through induction of p21 in a phospho-CAMKII-dependent pathway. Surprisingly, in spite of showing features of senescence following treatment with PLX4720 and ionizing radiation, Wnt5a-high melanoma cells were still able to invade and formed colonies in vivo [36]. As these initial studies demonstrate that Wnt5a can either induce or inhibit tumor progression through regulating cellular senescence, additional studies on the role of Wnt5a in senescence are needed in other cancers.

## 3. Wnt5a and Cancer Stem Cells

Cancer stem cells (CSC) are a small subpopulation of cells within the tumor that are characterized by self-renewal and high differentiation potential [38]. CSC exhibit phenotypic and functional characteristics that help to distinguish them from other cancer cells and they are believed to be responsible for tumor initiation, propagation, and relapse [38,39]. However, the CSC model remains controversial, as not all tumors express CSC markers [40].

In ovarian cancer, the Wnt5a receptor Ror1 was found to be highly expressed in high-grade and less-differentiated aggressive lesions and it was correlated with poor disease-free survival and overall survival [41,42]. Ovarian cancers that express high levels of Ror1 had gene-expression signatures associated with CSCs [43]. Furthermore, a direct correlation was observed between ALDH1, a marker of ovarian CSCs, formation of tumor spheroids in vitro, and Ror1 expression [43]. Treating primary ovarian patient-derived xenograft (PDX) tumor cells, which express high levels of Ror1 with an anti-Ror1 mAb (UC-961) inhibited spheroid formation and migration in vitro and engraftment in immune-deficient mice [43].

Wnt5a was also shown to up-regulate stem-like cell markers in nasopharyngeal carcinoma [44]. Stable knockdown of Wnt5a in S18 nasopharyngeal carcinoma cells significantly decreased the percentage of CD24^−^/CD44^+^ stem-like cells, while over-expression of Wnt5a in S26 nasopharyngeal carcinoma cells S26 significantly increased the percentage of CD24^−^/CD44^+^ cells [44]. Moreover in breast cancer, where Wnt5a is known to act as a tumor suppressor through inhibition of canonical Wnt signaling [45], a new perspective on the role of Wnt5a in inhibiting mammary carcinoma initiation was revealed [46]. Wnt5a produced by mammary luminal cells acts in a paracrine fashion, through binding to Ryk receptor on basal tumor initiating cells (TICs) and forms a complex with transforming growth factor β receptor 1 (TGFβR1), leading to the phosphorylation and activation of SMAD2, a signaling protein involved in regulating cell differentiation, and suppression of basal cells growth and tumor initiation [46]. As these data support a link between Wnt5a signaling and the regulation of CSC markers, Wnt5a signaling may represent a target for therapy that will also impact the CSC population.

## 4. Wnt5a and Chemotherapy Resistance

Chemotherapy resistance occurs when cancer cells acquire the ability to resist the effects of chemotherapeutic drugs [47]. The efficacy of chemotherapy is affected not only by the presence of the target, but also by presence or subsequent occurrence of mutations/genomic alterations that may alter the therapeutic response [47]. Chemotherapy resistance can arise due to several factors, including drug target alteration, re-activation of the targeted pathway, amplification of the oncogenic pathway, activation of parallel pathways and cell death inhibition [47].

Many studies have revealed that Wnt5a may drive chemotherapy resistance in different cancer types. Wnt5a was found to be highly upregulated in a subset of BRAF inhibitor (BRAFi)-resistant melanoma tumors and in BRAFi-resistant melanoma cells derived in vitro [48]. The elevated Wnt5a expression activated Wnt5a signaling through Fzd7 and Ryk receptors and induced PI3K/Akt signaling, leading to increased growth and therapeutic resistance in BRAFi-resistant cells as well as in patients who have relapsed on BRAF/MAPK inhibitors [48]. Another study showed a similar mechanism for Wnt5a-induced chemoresistance [49]. Prolonged exposure of colon cancer cells to increasing concentrations of butyrate, a histone deacetylase inhibitor (HDACi) that induces apoptosis in colon cancer cells, resulted in insensitivity to this agent as well as cross-resistance to structurally different HDACs [49]. Wnt5a was found to be upregulated in HDACi-resistant cells and this resistance mechanism was mediated by active Wnt5a signaling through Ror2 receptor, which activated Akt/PKB cell survival signaling [49]. Interestingly, recent studies show that in melanoma cells, Wnt5a activated protein kinase C (PKC), which phosphorylates STAT3, led to reduced expression of tumor associated-antigens, such as MART-1, PAX3, SOX10 and MITF, which are used as targets for immunotherapy, leading to impaired T-cell clearance of tumor cells [50]. This observation provides an alternative mechanism by which Wnt5a can impact therapy resistance.

Cancer multidrug resistance (MDR) is a mechanism wherein cancer cells develop resistance to structurally unrelated anticancer drugs [51]. While canonical Wnt signaling is correlated with promoting cancer MDR, mainly through active β-catenin and related transactivation activities regulating the cell cycle [52,53], there are other studies showing that non-canonical Wnt signaling can also induce MDR in several cancers through distinct mechanisms [49,54,55,56,57]. For example, Wnt5a signals through the transcription factor nuclear factor of activated T-cell C2 (NFATc2) to induce gemcitabine resistance and cell survival in pancreatic cancer in both in vivo and in vitro models [54]. Furthermore, ovarian cancer cell lines that have high levels of Wnt5a expression showed lower chemosensitivity to paclitaxel, oxaliplatin, 5-fluorouracil, epirubicin and etoposide [55,56]. Another study showed that the Wnt5a promotor is hypo-methylated in two multidrug-resistant cancer cell lines MES-SA/Dx5 and MCF7/ADR2 cells. The over-expression of Wnt5a in these two cell lines activated protein kinase A (PKA), which phosphorylates and inactivates GSK3β, leading to stabilization and activation of β-catenin, which in turn induces the expression of ATP-binding cassette sub-family B member (ABCB1), a key protein that is responsible for pumping therapeutic drugs out of cancer cells, leading to MDR. β-Catenin also activated the transcription of cyclin D1 and c-Myc, inducing cell proliferation and apoptosis resistance in MCF7/ADR2 and MES-SA/Dx5 cells under chemotherapy treatment [57]. In aggregate, these data suggest that Wnt5a signaling could be an attractive target to enhance cancer cell response to chemotherapeutic drugs.

## 5. Wnt5a and Tumor Microenvironment Cells

Wnt5a was also found to play a critical role in the interaction between tumor cells and the tumor microenvironment. Early studies showed that in non–small-cell lung cancer (NSCLC), intra-tumoral Wnt5a overexpression was correlated with increased expression of β-catenin and VEGF-A in stromal cells, suggesting tumor-stromal cross-talk [58]. Similarly, in phyllodes tumors, rare breast tumors of stromal origin, there was also a correlation between Wnt5a mRNA overexpression in the epithelium and strong nuclear β-catenin staining in stromal cells, suggesting that Wnt5a overexpression may be contributing to β-catenin upregulation in phyllodes tumors and that the epithelium may be driving the stromal proliferation of these tumors [59].

In gastric carcinoma, Wnt5a secreted by Cxcr4^+^ gastric innate lymphoid cells (ILCs) in the perivascular gastric stem cell niche enhanced the colony formation ability of Mist1^+^ gastric stem cells. Furthermore, ILCs-secreted Wnt5a activated RhoA, which enhanced sphere formation and promoted anchorage-independent cell growth of the gastric adenocarcinoma AGS cell line in a soft-agar assay [60]. Moreover, Wnt5a stimulation of E-cadherin-deficient Mist1^+^ stem cells, which are normally unable to survive in vitro upon E-cadherin deletion, induced prolonged survival through activation of RhoA in these cells [60]. In pancreatic ductal adenocarcinoma (PDAC), co-culture PDAC MiaPaCa2 cells with adipocytes induced MiaPaCa2 cells to secrete high levels of Wnt5a protein [61]. MiaPaCa2-secreted Wnt5a acted in an autocrine fashion on MiaPaCa2 cells and in a paracrine fashion on adipocytes cells, activating the c-JUN/AP1 pathway in both cell types. This resulted in reprograming of adipocytes to fibroblast-like cells that produce growth-promoting cytokines and provide lipids and other metabolites necessary to sustain tumor growth [61]. In intestinal cancer, enhanced stromal Wnt5a expression in mouse intestinal tumors promoted adhesion sites to form focally and stimulated directional migration and invasion of colon cancer cells [62]. This was correlated with local invasion and metastasis and thereby to colon cancer progression [62].

Another study showed that Wnt5a was highly expressed in prostate cancer cells that were derived from a bone metastasis site [63]. Moreover, bone marrow stromal cells secreted Wnt5a, which enhanced the migration of prostate cancer cells towards bone marrow stromal cells, suggesting a role of Wnt5a as a chemoattractant that induces prostate cancer metastasis to the bone [63]. Bone stromal cell derived-Wnt5a induced the expression of BMP-6 through activating the PKC/NFkB pathway in prostate cancer cells [64]. In turn, BMP-6 enhanced SMAD5 and β-catenin interaction, inducing prostate cell proliferation and resistance to androgen deprivation [64].

Using a co-culture experiment, depletion of Wnt5a in melanoma cells decreased endothelial cell branching while stimulation of endothelial cells with isolated melanoma cell exosomes, induced by incubation with rWnt5a, increased endothelial cell branching [65]. Another co-culture experiment showed that MCF-7: macrophage co-cultures induced the expression of macrophage Wnt5a that, in turn, activated AP-1/c-Jun in MCF-7 cells and enhanced migration and invasiveness [66]. Furthermore, Wnt5a was detected in tumor-associated macrophages in breast cancer biopsies [66]. Interestingly, Wnt5a was also shown to be constitutively expressed in microglia, the resident brain macrophage. Microglia induced breast cancer MCF-7 cell invasion in the brain in a Wnt5a-dependent manner, suggesting a mechanism whereby microglia may potentiate brain metastasis [67]. Together these studies suggest that Wnt5a signaling modulates the interaction between cancer cells and cells of the tumor microenvironment, contributing to disease progression.

## 6. Wnt5a and Cancer Associated-Inflammation

Inflammation is a hallmark of cancer that promotes cancer progression [68]; however a paradoxical pathological role for Wnt5a signaling in inflammation has been described, through promoting the secretion of both pro-inflammatory and anti-inflammatory cytokines [69,70,71]. Several studies suggest that similar effects may be observed in Wnt5a-related cancer-associated inflammation. For example, a recent study showed a Wnt5a immune-modulatory role in ovarian cancer cells [72]. Knockdown of Wnt5a in SKOV-3 cells suppressed the expression of granulocyte colony-stimulating factor (GCSF), granulocyte–macrophage colony-stimulating factor (GM-CSF), IL-1α, IL-13, and monocyte chemoattractant protein 3 (MCP-3) and enhanced the expression of RANTES/CCL5, cyclooxygenase-2 (COX-2) and inducible nitric oxide synthase (iNOS). This resulted in decreased cell migration [72]. Wnt5a induced gastric epithelial cells to produce IL-1β in an autocrine or paracrine manner, which in turn induced gastric epithelial cells to secrete MCP-1, a macrophage chemoattractant, leading to recruitment of macrophages to gastric mucosa and inducing gastric inflammation [73]. In melanoma, Wnt5a activated the Wnt/Ca^2+^ pathway, leading to activated CDC42, cytoskeletal changes, and the rapid release of exosomes containing the immunomodulatory cytokine IL-6 and the proangiogenic factors IL-8, VEGF and MMP2 [65]. In contrast, breast cancer patient tissue microarrays revealed a strong correlation between the expression of Wnt5a in malignant epithelial cells and the frequency of CD163^+^ anti-inflammatory tumor-associated macrophages [74].

In addition to the tumor cells, gliomas are comprised of many associated cell types including microglia, astrocytes and macrophages, which play a significant role in promoting tumor aggressiveness and progression [75]. Astrocyte-secreted Wnt5a was correlated with a pro-inflammatory microglia response, which is characterized by increased expression of iNOS, COX-2, cytokines, chemokines, enhanced invasive capacity and proliferation [76]. A recent study also showed that Wnt5a expression was positively correlated with the presence of microglia/monocytes in human gliomas and increased expression of human leukocyte antigens (HLA) genes, which are associated with a strong pro-inflammatory signature in the human glioma microenvironment. This indicates that Wnt5a, at least in part, is responsible for the characteristic inflammatory profile of the glioma [77]. While these studies highlight a significant role for Wnt5a in modulating cancer associated inflammation, additional studies are warranted.

## 7. Wnt5a and EMT

The epithelial-mesenchymal transition (EMT) is a developmental process in which epithelial cells acquire mesenchymal characteristics that change their morphology and enhance their migratory capacity [78]. In cancer, EMT is thought to facilitate the detachment of cancer cells from the primary tumor to enable their infiltration into surrounding tissues [79]. Triggering EMT in normal development and in cancer is associated with activation of several signaling pathways, including TGF-β, Wnt, EGF and FGF [79]. While it is well established that canonical Wnt/β-catenin signaling can be an activator of EMT [79], it was recently found that non-canonical Wnt5a signaling components such as PKC and JNK are also upregulated during EMT [80,81]. This led different studies to elucidate the role of Wnt5a in EMT and to explore the pathways activated downstream of Wnt5a. A novel pathway activated by Wnt5a has been shown to induce EMT in cell lines derived from late-stage mesenchymal-type hepatocellular carcinomas (HCC) and cancers of the breast, lung, and colon [82]. In these cell lines, Wnt5a binds to Fzd2 receptor and phosphorylates the transcription factor STAT3, a driver of EMT in diverse solid tumors, through the tyrosine kinase Fyn, leading to enhanced cell migration and metastasis. Knockdown of Fzd2 or treatment with an anti-Fzd2 antibody reduced HCC tumor growth and metastasis in a mouse xenograft model [82].

Moreover, another study on ovarian carcinoma showed that Wnt5a signaling through Ror1 and Ror2 receptors induced EMT and enhanced ovarian cancer cells migration and in trans-well assay [83]. Wnt5a also promoted EMT in nasopharyngeal cancers through activation of PKC and induction of Snail [44]. On the other hand, Wnt5a is a potential suppressor of EMT in gastric cancer, wherein epidermal growth factor (EGF) activation of EMT required suppression of Wnt5a expression through activating ADP-ribosylation factor (Arf6), which binds to and represses the Wnt5a promoter [84]. In colorectal cancer, where Wnt5a acts as a tumor suppressor and its loss is correlated with metastasis and poor survival rate, it was shown that Wnt5a enhanced epithelial characteristics and downregulated mesenchymal traits, suggesting a reversal of EMT [85]. Furthermore, transfecting colon cancer cells with Wnt5a led to downregulation of transcription factors considered key regulators of EMT, such as Twist and Zinc finger E-box-binding homeobox 1 (ZEB1), and induction of an epithelial phenotype by enhancing the expression of E-cadherin [85]. Wnt5a also reduced the expression of β-catenin and its target gene c-Myc [85]. Thus, Wnt5a inhibited EMT in part through antagonizing canonical Wnt/β-catenin signaling. In contrast, however, another study showed that Wnt5a expression stimulated directional migration and invasion of colon cancer cells and was correlated with poor prognosis [62]. Further research is needed clarify this apparent discrepancy about the role of Wnt5a signaling in colon cancer.

## 8. Wnt5a, Cell Migration, and Cell Invasion

Cell migration is a process that involves reorganization of the cytoskeleton, formation of protrusions, establishment of adhesive contacts at the leading edge, and cell contraction and detachment at the trailing edge [86]. In several cancers, Wnt5a was found to promote cell migration, thereby enhancing cancer cell invasion. For example, in nasopharyngeal cancer (NPC), overexpression of Wnt5a in S26 cells significantly induced migration and invasion while stable knockdown of Wnt5a in S18 cells significantly decreased migration and invasion [44]. Another study showed that recombinant Wnt5a protein induced migration and invasion in SCC9 and SCC25 oral squamous carcinoma cell lines in a wound healing assay, through activation of the non-canonical Ca^2+^/PKC pathway. These effects were blocked with the Wnt5a inhibitor peptide Box5 [87]. Wnt5a is highly expressed in gastric carcinoma wherein it has been shown to activate the small GTP-binding protein Rac, which in turn activated focal adhesion kinase (FAK) and paxillin, main regulators of cell migration [12]. Moreover, another study showed that Wnt5a induced gastric cancer migration and invasion through binding to Fzd2 and Ror2, leading to their internalization in a clathrin-dependent manner. This internalization was necessary for Wnt5a-dependent activation of Rac1 and expression of lamininγ2 [88]. Furthermore, an anti-Wnt5a antibody inhibited liver metastasis of gastric cancer cells in vivo [88].

Wnt5a is involved in regulating melanoma cell orientation with respect to the surrounding tissue, polarity, and directional movement in response to positional cues from chemokine gradients, through activating the small guanosine triphosphatases Rab4 and RhoB [89]. This in turn leads to the recruitment of actin, myosin IIB, Fzd3, and melanoma cell adhesion molecule (MCAM) into an intracellular structure of melanoma cells asymmetrically at the cell periphery, where it triggers membrane contractility and nuclear movement in the direction of membrane retraction [89]. Increased Wnt5a signaling through Fzd5 in melanoma cells enhanced in vitro motility and invasion through activating PKC that is important in regulating cell cytoskeleton, adhesion, and motility [11]. The Wnt5a-derived hexapeptide Box5, which inhibits Wnt5a signaling, reduced Wnt5a-induced melanoma cell motility [90]. Furthermore, Wnt5a also disrupts the N-cadherin and β-catenin complexes by binding to Fzd4/LRP6 receptor complex and activating ARF6, thereby increasing the pool of free β-catenin in the cytoplasm, which translocates to the nucleus, enhances β-catenin-mediated transcription, and stimulates melanoma cells invasion [91]. A distinct mechanism for Wnt5a induction of melanoma cell motility is through binding to Ror2 and activating of Wnt/Ca^+2^ signaling, leading to activation of calpain-1, a calcium-dependent non-lysosomal cysteine protease. Active calpain-1 cleaved filamin A, which resulted in melanoma cells cytoskeletal changes and enhanced motility [92].

Wnt5a is upregulated in prostate cancer cells compared to normal prostate cells due to hypo-methylation of the gene promoter region [93]. This Wnt5a upregulation led to activation of the Wnt/Ca^2+^ pathway, activating CaMKII which caused a major reorganization of cytoskeleton in cancer cells by decreasing the length and frequency of filopodia-like actin structures and enhancing cell motility [93]. Another study on prostate cancer showed that Wnt5a bound to Fzd2 and Ror2 and activated JNK signaling, leading to expression of matrix metalloproteinase 1 (MMP1) through enhancing the recruitment of JunD to the AP-1 site of MMP1 promoter region, thereby inducing cell migration and invasion [94]. Furthermore, Wnt5a enhanced the vasculogenic mimicry, EMT, motility and invasiveness of ovarian cancer cells in a PKCα-dependent manner [95].

Chronic lymphocytic leukemia (CCL) cells are induced to migrate by Wnt5a in a chemokine gradient manner, which is important in regulating chemotaxis and trans-endothelial migration of CLL cells, apparently through upregulating PCP components including Vangl2, Prickle1, CK1ε, Dvl, and Celsr1 [96]. On the contrary, ectopic expression of Wnt5a inhibited clonogenicity and motility of esophageal squamous cell carcinoma (ESCC) cells through antagonizing the Wnt/β-catenin pathway [97]. Together these data support a role for Wnt5a in tumor cell migration, through activation of distinct signaling pathways. These results suggest that strategies to block Wnt5a-induced invasion will likely be cell type specific and dependent on the differential expression of Wnt5a receptors.

## 9. Wnt5a and Metastasis

Many studies have revealed a strong correlation between Wnt5a protein expression level and cancer metastasis. For instance, Wnt5a protein level was high in pulmonary metastases from nasopharyngeal cancer (NPC) patients. *WNT5A* mRNA was also elevated in hepatic metastases from nasopharyngeal cancer and was correlated with poor survival [44]. Moreover*,* in vivo animal experiments showed that administration of recombinant Wnt5a protein significantly induced lung metastasis of NPC cells [44]. In gastric cancer, Wnt5a induced the expression of the extracellular matrix protein lamininγ2, a subunit of the extracellular matrix laminin 5 protein that constitutes the epithelial basement membrane, through activating Dvl-associating protein with a high frequency of leucine residues (Daple), which in turn activates Rac and JNK to promote invasion and metastasis [98]. In melanoma, Wnt5a expression was correlated strongly with both survival and time to metastasis. The expression of Wnt5a increased with cutaneous melanoma progression and expression was shown to be a risk factor for outcome [99]. Wnt5a expression was also upregulated in cervical cancer tissues compared with adjacent normal cervix and it was positively correlated with both lymph node metastasis and recurrence [100]. Wnt5a/JNK signaling may regulate the microvascular and peritoneal invasion and lymph node metastasis of pancreatic adenocarcinoma through activating paxillin [101]. Overall, these data suggest that inhibition of Wnt5a signaling may represent a strategy to reduce metastasis in many cancer types.

## 10. Wnt5a and Proliferation

While Wnt5a expression contributes to tumor aggressiveness by inducing cancer cell invasion and metastasis, it can also enhance cell proliferation in some types of cancer. Wnt5a predominantly activates β-catenin-independent signaling through induction of clathrin-dependent receptor-mediated endocytosis [102,103]. A recent study suggests that Wnt5a can also activate its downstream signaling in an endocytosis-independent mechanism that involves activation of Src family kinases (SFKs) and can induce cervical, lung, and esophageal cancer cell proliferation [104]. Indeed, using a blocking antibody that impairs Wnt5a-induced receptor-mediated endocytosis did not affect proliferation in these cells, even though invasion was inhibited [104]. Moreover, overexpression of the Wnt5a protein in the pancreatic cancer cell lines PANC-1 and BXPC-3 induced tumor cell proliferation and decreased apoptosis in an orthotopic nude mouse model [105].

Furthermore, Wnt5a can induce proliferation in chronic lymphocytic leukemia (CLL) cells through a distinct mechanism. In CLL cells, Wnt5a induces oligomerization of Ror1 and Ror2, which in turn recruits guanine exchange factors (GEFs), thereby activating Rac1, which is associated with enhanced leukemia cell proliferation [106]. In non-small-cell lung cancer (NSCLC), overexpression of Wnt5a produced more aggressive cancer, especially in squamous cell carcinomas wherein it was significantly correlated with the Ki-67 proliferation index [56]. Wnt5a levels were higher in human glioblastoma (GMB) than in normal brain tissue and low-grade astrocytoma [107]. Correlative in vitro studies showed that the overexpression of Wnt5a enhanced the proliferation of the GMB cell lines GBM-05 and U87MG [107].

On the other hand, Wnt5a overexpression induced prostate cancer cell apoptosis, reduced proliferation, diminished tumor growth and prevented the establishment of bone lesions in vivo model [108]. Moreover, Wnt5a was shown to signal through a non-canonical Wnt/Ca^+2^ pathway to suppress cyclin D1 expression and inhibit B cell proliferation in a cell-autonomous manner. This is consistent with the observation that loss of Wnt5a expression leads to formation of hematopoietic malignancies, such as myeloid leukemias and B cell lymphomas [109]. In intestinal cancer, overexpression of Wnt5a inhibited cell proliferation and diminished tumorigenicity in nude mice models through down-regulating the expression of c-Myc [85]. Moreover, Wnt5a induced cell apoptosis through activating CaMK II, downregulating Bcl-2 expression and enhancing Bax expression [85]. Overall, these data suggest that Wnt5a regulation of cancer cell proliferation is dependent on the context of downstream effectors.

## 11. Wnt5a and Cancer Cell Metabolism

Tumor cells undergo metabolic reprogramming to provide more energy to the rapidly proliferating tumor cells. One of main metabolic features of cancer cells is the elevated level of aerobic glycolysis, which results in increased glucose consumption and lactate production, through coordinated upregulation of glucose transporters and glycolysis enzymes by oncogenes such as c-Myc and protein kinase B (Akt) [110,111]. The Akt pathway is a very important regulator of cancer cell metabolic reprogramming through activating the serine/threonine kinase mTOR, which is a key regulator of protein synthesis, cell growth, and cellular metabolism [112]. mTOR signaling activates aerobic glycolysis in cancer cells through stimulating glycolytic enzymes activity such as hexokinase and phosphofructose kinase [111]. While the involvement of canonical Wnt signaling in regulating normal and cancer cell metabolism is well studied [113], it was recently shown for the first time that non-canonical Wnt signaling can induce metabolic reprogramming in melanoma and breast cancer [114]. In melanoma cells, Wnt5a stimulates Akt-mTORC1 signaling through the Wnt/Ca^+2^ pathway. Wnt5a-activated Akt-mTORC1 signaling increased the levels of lactate dehydrogenase (LDH), an enzyme required for the formation of lactate, and production of NAD^+^ required for the action of glyceraldehyde-3-phosphate dehydrogenase, thus supporting glycolysis. Moreover, LDH was shown to be expressed at high levels in melanoma and correlate with poor prognosis [115]. This suggests a new oncogenic role for Wnt5a in inducing melanoma aggressiveness. In contrast, Wnt5a enhanced mitochondrial-mediated oxidative phosphorylation respiration in breast cancer cells [114]. These two reports highlight a new role for Wnt5a signaling as a regulator of cancer cell metabolism.

## 12. Wnt5a in Ovarian Cancer

**Figure 2:**
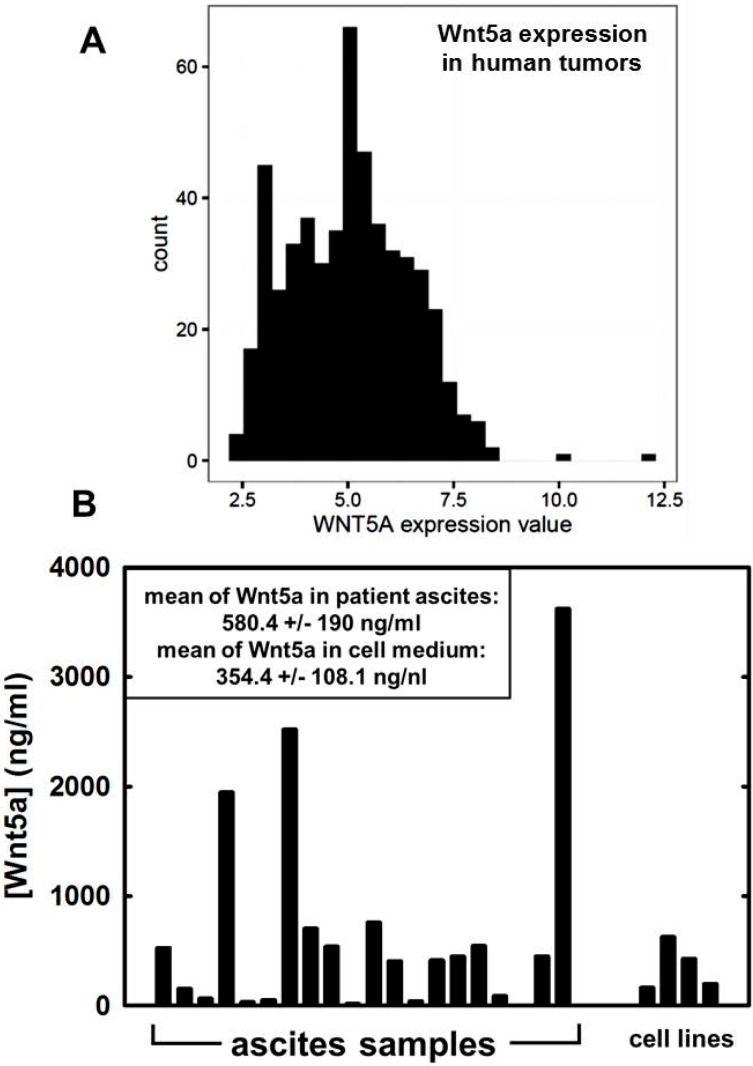
Wnt5a expression in human ovarian cancer. (**A**) Summary of *WNT5A* expression in human ovarian cancer. Data are obtained from The Cancer Genome Atlas (TCGA) and represent 583 ovarian cancer samples. Gene expression was measured using the Affymetrix microarray hgu133a. Data were normalized with gcrma and relative expression values (y-axis) are on a log2 scale [121]. Counts (x-axis) indicates the number of patients with a specific expression value x; (**B**) Analysis of Wnt5a protein levels in human ovarian cancer ascites. Ascites fluid was obtained from women with ovarian cancer. After centrifugation to remove cellular components, WNT5A levels were determined by ELISA using 100 uL of ascites. The mean concentration of WNT5A in ascites (*n* = 20) is 584.4 ± 190 ng/mL. Similar levels are expressed by cultured ovarian cancer cell lines (OvCa 429, OvCa433, DOV13, SKOV-i.p.; mean 354.4 ± 108.1 ng/mL).

Although Wnt signaling has not been extensively investigated in ovarian cancer, recent data support a role for both the canonical and non-canonical Wnt pathways in ovarian cancer pathology. An early study of primary ovarian tumors (*n* = 130) compared to normal ovarian surface or fallopian tube epithelium (*n* = 31 and 28, respectively) showed lower Wnt5a expression relative to normal tissue. Furthermore, low Wnt5a expression was correlated with both tumor stage and overall survival, suggesting that loss of Wnt5a is predictive of poor outcome [34]. In contrast, high Wnt5a staining in ovarian cancer relative to normal ovary was found to correlate with poor prognosis in two related studies [55,116]. More recently, analysis of a large cohort (*n* = 623) of patients found upregulation of Wnt5a expression in all major histotypes relative to benign controls [83]. Analysis of data obtained from The Cancer Genome Atlas (TCGA) support these findings (Figure 2A). Examination of data from 583 ovarian tumors show that Wnt5a is highly expressed. Furthermore, Wnt5a protein is prevalent in ascites obtained from women with ovarian cancer (Figure 2B), [117], suggesting a contribution to the ovarian tumor microenvironment. This is consistent with the observation that a significant correlation was found between Wnt5a expression and metastasis (*n* = 43) [95]. It is interesting to note that activating mutations in the canonical Wnt pathway are rare in ovarian cancer, with the exception of endometrioid ovarian cancers [118]. However, recent studies highlight a number of mechanisms for activation of canonical Wnt signaling in ovarian cancer through multivalent integrin engagement, changes in matrix rigidity, and via bioactive lipid signaling pathways [117,119,120]. Thus, additional research is needed to delineate the roles of both canonical and non-canonical Wnt signaling in ovarian cancer development, progression and metastasis.

## 13. Additional Considerations

As summarized above, the role of Wnt5a in cancer is varied and complex. A number of diverse biologic processes are regulated downstream of Wnt5a that may contribute to tumor progression (Figure 1). Additional research on tissue-specific expression of specific ligands, receptors, and co-receptors is needed to elucidate molecular mechanisms that drive altered cellular behavior. Careful consideration of cells in the tumor microenvironment including fibroblasts, adipocytes, and inflammatory cells is also warranted. Interesting recent findings of a role for Wnt5a in regulation of cancer cell metabolism also merits further investigation. A more detailed understanding of the complex cross-talk between Wnt5a, its receptors and co-receptors in the tumor microenvironment will enable the development of inhibitors to block aberrant signaling and thereby favorably impact patient survival.
